# Observation of novel charge ordering and spin reorientation in perovskite oxide PbFeO_3_

**DOI:** 10.1038/s41467-021-22064-9

**Published:** 2021-03-26

**Authors:** Xubin Ye, Jianfa Zhao, Hena Das, Denis Sheptyakov, Junye Yang, Yuki Sakai, Hajime Hojo, Zhehong Liu, Long Zhou, Lipeng Cao, Takumi Nishikubo, Shogo Wakazaki, Cheng Dong, Xiao Wang, Zhiwei Hu, Hong-Ji Lin, Chien-Te Chen, Christoph Sahle, Anna Efiminko, Huibo Cao, Stuart Calder, Ko Mibu, Michel Kenzelmann, Liu Hao Tjeng, Runze Yu, Masaki Azuma, Changqing Jin, Youwen Long

**Affiliations:** 1 Beijing National Laboratory for Condensed Matter Physics, Institute of Physics, Chinese Academy of Sciences, Beijing, China; 2 School of Physical Sciences, University of Chinese Academy of Sciences, Beijing, China; 3 Laboratory for Materials and Structures, Tokyo Institute of Technology, Yokohama, Kanagawa Japan; 4 Tokyo Tech World Research Hub Initiative (WRHI), Institute of Innovative Research, Tokyo Institute of Technology, Yokohama, Kanagawa Japan; 5 Laboratory for Neutron Scattering and Imaging, Paul Scherrer Institut, Villigen, Switzerland; 6 Kanagawa Institute of Industrial Science and Technology, Ebina, Japan; 7 Department of Advanced Materials and Engineering, Faculty of Engineering Sciences, Kyushu University, Kasuga, Japan; 8 Max-Planck Institute for Chemical Physics of Solids, Dresden, Germany; 9 National Synchrotron Radiation Research Center, Hsinchu, Taiwan, ROC; 10 European Synchrotron Radiation Facility, Grenoble, France; 11 Neutron Scattering Division, Oak Ridge National Laboratory, Oak Ridge, TN USA; 12 Graduate School of Engineering, Nagoya Institute of Technology, Nagoya, Japan; 13 Songshan Lake Materials Laboratory, Dongguan, Guangdong China

**Keywords:** Magnetic properties and materials, Structure of solids and liquids

## Abstract

Pb*M*O_3_ (*M* = 3*d* transition metals) family shows systematic variations in charge distribution and intriguing physical properties due to its delicate energy balance between Pb 6*s* and transition metal 3*d* orbitals. However, the detailed structure and physical properties of PbFeO_3_ remain unclear. Herein, we reveal that PbFeO_3_ crystallizes into an unusual 2*a*_p_ × 6*a*_p_ × 2*a*_p_ orthorhombic perovskite super unit cell with space group *Cmcm*. The distinctive crystal construction and valence distribution of Pb^2+^_0.5_Pb^4+^_0.5_FeO_3_ lead to a long range charge ordering of the -A-B-B- type of the layers with two different oxidation states of Pb (Pb^2+^ and Pb^4+^) in them. A weak ferromagnetic transition with canted antiferromagnetic spins along the *a*-axis is found to occur at 600 K. In addition, decreasing the temperature causes a spin reorientation transition towards a collinear antiferromagnetic structure with spin moments along the *b*-axis near 418 K. Our theoretical investigations reveal that the peculiar charge ordering of Pb generates two Fe^3+^ magnetic sublattices with competing anisotropic energies, giving rise to the spin reorientation at such a high critical temperature.

## Abstract (web summary)

PbFeO_3_ is part of a family of lead based perovskites with many intriguing properties; however, difficulties in synthesis have hampered investigation. Here, the authors present a detailed study of the structure of PbFeO_3_ observing unique charge ordering and spin orientation among the constituent ions.

## Introduction

Transition metal perovskite oxides (general formula: *AB*O_3_) display a variety of desirable electronic and magnetic properties, such as high-temperature superconductivity^1^, colossal magnetoresistance^2,3^, metal–insulator transition^4^, multiferroicity^5–7^, and electrocatalysis^8^. *AM*O_3_ (*A* = Pb/Bi) perovskite oxides are typical examples of charge degrees of freedom at the *A* site depending on 6*s*^0^ (Pb^4+^, Bi^5+^) and 6*s*^2^ (Pb^2+^, Bi^3+^) electron configurations for the prohibition of the 6*s*^1^ configuration^9–11^. For example, in Bi-based systems, BiCrO_3_ through BiCoO_3_ are all Bi^3+^*M*^3+^O_3_; however, BiNiO_3_ has an unusual valence state Bi^3+^_0.5_Bi^5+^_0.5_Ni^2+^O_3_ with ordered Bi^3+^ and Bi^5+^ charge states^9–15^.

In Pb-based systems, as the *d* level of the transition metal becomes deeper, different crystal structures and systematic charge distribution changes are observed^16–22^. Divalent lead appears in tetragonal PbTiO_3_ and PbVO_3_^17^. However, the disordered coexistence of Pb^2+^ and Pb^4+^ states (charge glass) occurs in PbCrO_3_ (Pb^3+^Cr^3+^O_3_ on average), where a simultaneous insulator-to-metal transition and a large volume collapse arise from the melting of Pb charge glass and Pb–Cr charge transfer upon pressurizing to 2.5 GPa^18^. More interestingly, a 1:3 ordered Pb^2+^ and Pb^4+^ and a 1:1 ordered Co^2+^ and Co^3+^ have been observed in PbCoO_3_ with the charge format of Pb^2+^Pb^4+^_3_Co^2+^_2_Co^3+^_2_O_12_ (Pb^3.5+^Co^2.5+^O_3_ on average)^19,20^. Moreover, a pressure-induced spin-state transition and Pb–Co intermetallic charge transfer have been discovered in this compound^19,20^. For PbNiO_3_, the electronic configuration is Pb^4+^Ni^2+^O_3_ in the presence of a single-valence Pb^4+^ state^21^. Currently, in Pb*M*O_3_ systems, only PbMnO_3_ and PbFeO_3_ remain elusive. Herein, we focus on PbFeO_3_, which has been reported by Tsuchiya et al. in 2007^22^. However, the difficulties in the synthesis of samples and in resolving the crystal structure have inhibited investigation of its structure and physical properties.

Alongside the intriguing charge properties at the Pb site, the spin degree of freedom at the *M*-site in Pb*M*O_3_ systems has also received significant attention. For example, a pressure-induced high-spin to low-spin-state transition of Co^2+^ accompanied by an unusual increase in the resistance in PbCoO_3_ has been reported recently^19^. The perovskite family of *R*FeO_3_ (*R* = rare earth) exhibits various spin-related properties such as multiferroicity^23–25^, laser-induced ultrafast spin reorientation (SR)^26,27^, and ultrafast photomagnetic recording^28^, and it is anticipated that the evolution of magnetism and spin structure of PbFeO_3_ as a function of temperature, magnetic field or pressure will be similarly rich. A characteristic feature of *R*FeO_3_ is the presence of two magnetic sublattices, i.e., the *R*^3+^ sublattice and Fe^3+^ sublattice^29–32^, which generate three types of competitive exchange interactions (Fe–Fe, *R*–Fe, and *R*–*R*). The strongest Fe–Fe interaction induces a canted antiferromagnetic (AFM) ordering of Fe^3+^ (*S* = 5/2) spins below the Néel temperature *T*_N_ = 600–740 K with a Néel vector *G*_*x*_ and a weak ferromagnetic (FM) vector *F*_*z*_. This type of spin structure is energetically preferred for Fe^3+^ moments and occurs in almost all Fe^3+^O_6_ perovskite-type frameworks^25,30,33–35^. Furthermore, weak *R*–*R* interactions can result in AFM ordering in the *R* sublattice at a low temperature (<10 K). One of the most interesting phenomena in *R*FeO_3_ is the SR induced by temperature and/or magnetic field, where the alignment of Fe^3+^ spin moments changes from one crystal direction to another. As the Fe spin reorientation is regarded to be closely related to anisotropic *R*–Fe magnetic exchange interactions, the current PbFeO_3_ with nonmagnetic *A*-site Pb ions may provide a new avenue for understanding the distinct underlying mechanism of SR transition.

In this study, comprehensive investigations based on synchrotron X-ray diffraction (SXRD), neutron powder diffraction (NPD), electron diffraction (ED), hard X-ray photoemission spectroscopy (HAXPES), soft X-ray absorption spectroscopy (XAS), Mössbauer spectroscopy, characterization of magnetic and electrical properties, and density functional theory (DFT) calculations were performed to examine the structure, charge state, and magnetic properties of PbFeO_3_. We discovered that PbFeO_3_ crystallized into a *Cmcm* space group with a new charge ordering of a unique -A-B-B- type, whereby in the direction of the layers stacking, one layer composed by Pb^2+^ is interleaved by two layers built up from a mixture of Pb^4+^ and Pb^2+^ in a 3:1 ratio. Moreover, a high-temperature weak FM transition and subsequently a spin reorientation transition occurred at ~600 and 418 K, respectively. Related mechanisms are proposed to explain the SR of PbFeO_3_.

## Results

### Crystal structure of PbFeO_3_

**Fig. 1:**
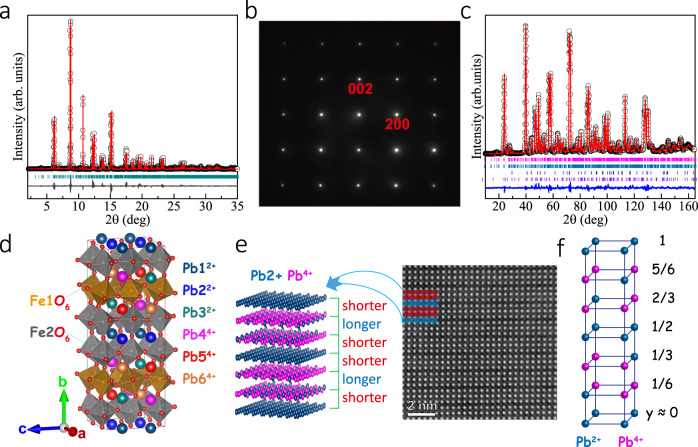
Synthesis and crystal structure characterizations of PbFeO_3_. **a** Rietveld refinement for SXRD pattern recorded at room temperature for PbFeO_3_. Observed (black circles), calculated (red line), and difference (gray line) values are shown. The allowed Bragg positions in *Cmcm* symmetry are indicated by ticks (dark cyan). **b** ED patterns along [010] pseudocubic zone axis at RT. **c** Rieveld refinement for NPD pattern at 300 K for PbFeO_3_. Observed (black circles), calculated (red line), and difference (blue) values are shown. Bragg positions of PbFeO_3_ are indicated by ticks (dark cyan), which correspond to the allowed nuclear (magenta) and magnetic (dark cyan) Bragg peaks of PbFeO_3_; and allowed nuclear (navy) and magnetic (violet) Bragg reflections of the impurity phase Fe_2_O_3_ (~5 wt%). **d** Crystal structure of PbFeO_3_. **e** Left part: illustration of Pb modulations; Right part: HAADF image along [001] pseudocubic zone axis of PbFeO_3_. Distances for the bright spots, which are the locations of Pb, indicate a modulation with a shorter–longer–shorter pattern. **f** Sketch of unique -A-B-B- type of charge ordering of PbFeO_3_ composed of two types of differently charged layers.

According to a previous report^22^, the sample quality of PbFeO_3_ is extremely sensitive to the synthesis conditions. We carefully optimized the synthesis pressure and temperature, and finally obtained a nearly single phase by synthesizing at 8 GPa and 1423 K for 30 min. The SXRD pattern of PbFeO_3_ is shown in Fig. 1a. Except for a tiny Fe_2_O_3_ impurity phase, all the diffraction peaks can be indexed based on an unusual 2*a*_p_ × 6*a*_p_ × 2*a*_p_ orthorhombic perovskite super unit cell, where *a*_p_ refers to the pseudolattice parameter of a cubic *AB*O_3_ perovskite subcell. To identify the reflection conditions as well as possible space groups, we performed an ED experiment. ED patterns indicate that PbFeO_3_ has an orthorhombic cell with a 2*a*_p_ × 6*a*_p_ × 2*a*_p_ superlattice (see Fig. 1b and Supplementary Fig. S1). The reflection conditions are 0*kl* (*k* = 2*n*), *h*0*l* (*h*, *l* = 2*n*), *hk*0 (*h* + *k* = 2*n*), *h*00 (*h* = 2*n*), 0*k*0 (*k* = 2*n*), and 00 *l* (*l* = 2*n*), which are consistent with the space groups of *Cmc*2_1_ (No. 36), *C*2*cm* (No. 40), and *Cmcm* (No. 63). We determined the final crystal structure using a Rietveld refinement of the SXRD pattern based on the primary structural models suggested by the ED. In comparison, we discovered that the most reliable structure model was *Cmcm* (see Fig. 1a), as reducing the symmetry cannot improve the quality of the fit any more. Since X-rays are not sensitive to the light element of oxygen, we perform NPD to precisely determine the oxygen position. The Rietveld refinement results of NPD data collected at 300 K are illustrated in Fig. 1c. We observed a *G*-type AFM structure at 300 K, which will be discussed later. In the crystal symmetry of *Cmcm*, Pb atoms occupied six special Wyckoff positions 4*c* (0, *y*, 0.25), Fe atoms occupied two special positions 8*d* (0.25, 0.25, 0) and 16*h* (*x*, *y*, *z*), whereas O atoms occupied one 16 *h* (*x*, *y*, *z*) site, three different 8*f* (0, *y*, *z*) sites, three different 8*g* (*x*, *y*, 0.25) sites, and one 8*e* (*x*, 0, 0) site. Table 1 and Supplementary Table S1 list the refined structure parameters, including the detailed lattice constants and atomic positions. The bond lengths and the results of bond valence sum (BVS) calculations for the NPD and SXRD data are listed in Table 2 and Supplementary Table S2, respectively. The obtained lattice parameters were *a* = 7.89945(15) Å, *b* = 23.46820(45) Å, and *c* = 7.73406(15) Å (corresponding to perovskite cell parameters *a*_p_ of 3.94973, 3.91137, and 3.86703 Å along with directions *a*, *b*, and *c*, respectively). As shown in Fig. 1d, each Fe atom is coordinated by six ligand O atoms with Fe–O distances varying from 1.95 to 2.14 Å, forming a perovskite-type FeO_6_ octahedron framework (see Fig. 1d). Moreover, the structure refinement results demonstrate that the Pb layers perpendicular to the *b* axis show a –shorter–longer–shorter– stacking with an interlayer distance of 3.8078 Å, 4.1186 Å, and 3.8078 Å. Figure 1e shows the high-angle annular dark-field (HAADF) image of PbFeO_3_. It is clear that the distances for the bright spots, which are the locations of the Pb layers, show a modulation similar to the –shorter–longer–shorter– pattern mentioned above. This result further confirms the reliability of the crystal structure determined from the ED, SXRD, and NPD data.

**Table 1:** Crystallographic parameters of PbFeO_3_ refined from NPD pattern at RT^a^.

Atom	Site	*x*	*y*	*z*	100×*B*_iso_ (Å)
Pb1	4*c*	0	0.9966 (2)	0.25	1.04 (2)
Pb2	4*c*	0	0.4999 (2)	0.25	1.04 (2)
Pb3	4*c*	0	0.8284 (2)	0.25	1.04 (2)
Pb4	4*c*	0	0.1540 (2)	0.25	1.04 (2)
Pb5	4*c*	0	0.3351 (2)	0.25	1.04 (2)
Pb6	4*c*	0	0.6585 (2)	0.25	1.04 (2)
Fe1	8*d*	0.25	0.25	0	0.52 (2)
Fe2	16*h*	0.2528 (4)	0.5841 (1)	0.9981 (3)	0.52 (2)
O1	8*g*	0.3034 (5)	0.7742 (2)	0.25	0.941 (2)
O2	16*h*	0.3094 (3)	0.6712 (1)	0.0549 (5)	0.941 (2)
O3	8*f*	0	0.2706 (2)	0.9374 (6)	0.941 (2)
O4	8*e*	0.2781 (5)	0	0	0.941 (2)
O5	8*f*	0	0.0768 (2)	0.0609 (5)	0.941 (2)
O6	8*g*	0.1769 (5)	0.5870 (2)	0.25	0.941 (2)
O7	8*f*	0	0.6093 (2)	0.9619 (5)	0.941 (2)
O8	8*g*	0.2781 (5)	0.4054 (2)	0.25	0.941 (2)

^a^Space group *Cmcm* (No. 63), *Z* = 24. *a* = 7.89945 (15) Å, *b* = 23.46820 (45) Å, *c* = 7.73406 (15) Å, *V* = 1433.727 (4) Å^3^. *R*_wp_ = 5.81%, *R*_p_ = 5.27%.

**Table 2:** Pb–O and Fe–O bond lengths and BVSs^a^ for PbFeO_3_ refined from NPD pattern at RT.

	Distance (Å)		Average bond length (Å)	BVS
Fe1–O	2.090 (1)	×2	2.035	2.82
	2.059 (1)	×2		
	1.955 (3)	×2		
Fe2–O	1.989 (2)		2.039	2.80
	2.040 (2)			
	2.101 (2)			
	2.137 (3)			
	1.945 (2)			
	2.020 (2)			
Pb1–O	2.385 (5)	×2	2.793	1.94
	2.766 (5)	×2		
	2.928 (3)	×4		
	2.958 (4)	×2		
Pb2–O	2.475 (5)	×2	2.772	2.08
	2.610 (3)	×4		
	3.043 (5)	×2		
	3.123 (5)	×2		
Pb3–O	2.517 (5)	×2	2.806	2.06
	2.713 (4)	×2		
	2.739 (5)	×2		
	2.798 (2)	×4		
	3.275 (3)	×2		
Pb4–O	2.169 (2)	×4	2.577	3.99
	2.328 (5)	×2		
	2.998 (4)	×2		
	3.221 (4)	×2		
Pb5–O	2.095 (5)	×2	2.451	3.92
	2.110 (5)	×2		
	2.747(4)	×2		
	2.852 (5)	×2		
Pb6–O	2.184 (5)	×2	2.535	3.62
	2.206 (5)	×2		
	2.510 (4)	×2		
	2.888 (2)	×4		

^a^*V*_*i*_  = ∑_*j*_*S*_*ij*_, *S*_*ij*_ = exp{(*r*_0_-*r*_*ij*_)/0.37}. Values calculated using *r*_0_ = 2.112 for Pb^2+^, 2.042 for Pb^4+^, and 1.751 for Fe^3+^.

### Charge-order structure of PbFeO_3_

The BVS calculations (see Table 2) reveal that the lead atoms, located at the three positions (Pb1, Pb2, and Pb3), show a valence state close to +2, but the other three (Pb4, Pb5, and Pb6) provide valence sums corresponding to a valence state close to +4. However, Fe always shows a +3 state regardless of its atomic positions. These features indicate that the valence distribution of PbFeO_3_ should be Pb^2+^_0.5_Pb^4+^_0.5_Fe^3+^O_3_. Based on the charge distribution in the *ac* plane with different *y*-axis values, Pb^2+^ and Pb^4+^ show a unique long-range charge ordering, as shown in Fig. 1f. Specifically, there exist two types of layers with respect to the estimated valence states of Pb. Within each unit cell repetition period along the crystallographic *b* axis, two identically charged layers consisting of Pb1 and Pb2 atoms characterized by a Pb^2+^ charge state are located at *y* ≈ 0 and 1/2. In addition, four differently charged layers, each built up by three Pb^4+^ atoms: Pb4, Pb5, and Pb6 and just one Pb^2+^ charged Pb3 atom located at *y* ≈1/6, 1/3, 2/3, and 5/6, respectively. This 3:1 ratio of the Pb^4+^ and Pb^2+^ ions provided an average oxidation state +3.5 for Pb atoms in these layers. Therefore, a peculiar -A-B-B- charge ordering, i.e., one layer with exclusive Pb^2+^ oxidation state (A) is followed by two layers (B) with an average oxidation state of Pb^3.5+^, was realized in PbFeO_3_. This unprecedented charge ordering renders PbFeO_3_ unique in all the reported charge-order perovskite oxides^36,37^, resulting in the formation of a 2*a*_p_ × 6*a*_p_ ×  2*a*_p_ perovskite supercell. Neither the melting of Pb charge ordering nor the valence and spin-state changes of Fe were observed when the sample was heated to temperatures near its decomposition temperature of approximately 730 K (Supplementary Fig. S2) or cooled to a low temperature down to 10 K, as confirmed by temperature-dependent XAS (see Supplementary Fig. S3). Other charge-order compounds in *AM*O_3_ perovskite systems, such as BiNiO_3_, PbCrO_3_, and PbCoO_3_ as mentioned above^16,18,19^, remained unchanged upon cooling or heating at ambient pressure; however, they exhibited pressure-induced intersite charge transfer transitions. Moreover, BiNiO_3_ with *R*, Pb, and Sb substitution for Bi or Fe substitution for Ni indicated a temperature-induced charge transfer accompanied by negative thermal expansion^15,38–43^. The investigation of current PbFeO_3_ under pressure and doping effects will be performed in our future study.

**Fig. 2:**
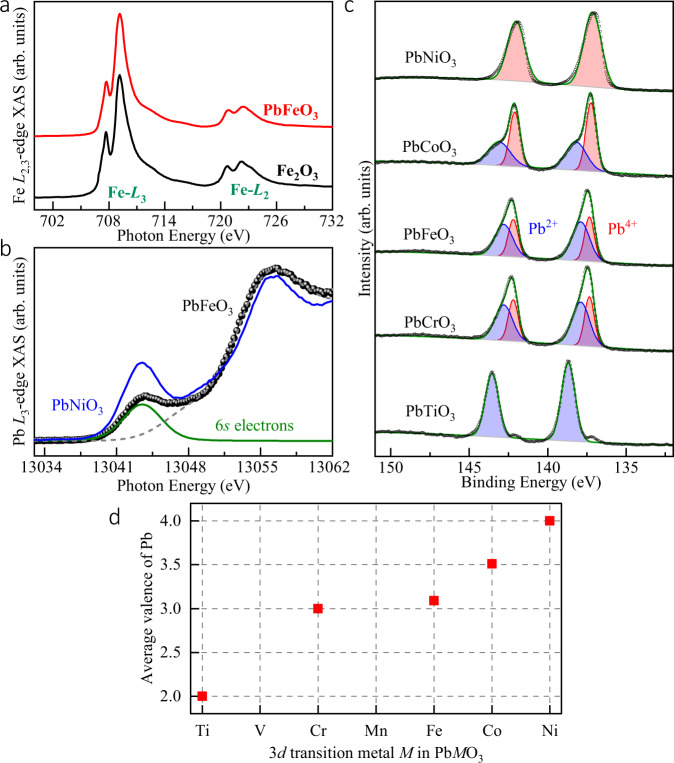
Valence-state characterization by XAS and HAXPES spectra. **a** XAS of Fe *L*_2,3_-edge of PbFeO_3_, and reference Fe_2_O_3_; **b** Pb *L*_3_-edge XANES of PbFeO_3_ (black circles) and PbNiO_3_ (blue curve) for comparison. The green curve in (**b**) shows the pre-edge peak 6*s* state of PbFeO_3_ after subtracting the background (dashed gray curve). **c** Pb-4*f* HAXPES results for PbTiO_3_, PbCrO_3_, PbFeO_3_, PbCoO_3_, and PbNiO_3_ at RT. Predominant of Pb^4+^ and Pb^2+^ are evident in the spectrum for PbFeO_3_. **d** Average Pb valence state calculated from area ratios of Pb^2+^ and Pb^4+^ components. PbTiO_3_, PbCrO_3_, and PbNiO_3_ are standard for Pb^2+^, Pb^3+^ (Pb^2+^_0.5_Pb^4+^_0.5_), and Pb^4+^, respectively.

The distinctive valence distribution of PbFeO_3_ can be further confirmed by Fe *L*_2,3_-edges XAS, Pb *L*_3_-edge XAS, and Pb-4*f* XPS. The sharp multiple spectral features at the 3*d* transition element *L*_2,3_-edges are extremely sensitive to the valence state^44,45^ and local environment^46,47^. The Fe *L*_2,3_-edge results of PbFeO_3_ are shown in Fig. 2a, together with Fe_2_O_3_ as a Fe^3+^ reference. Both of them show similar peak energy positions and spectral features, indicating the formation of the Fe^3+^ (3*d*^5^) state in PbFeO_3_, consistent with the BVS result. Considering that the Pb *L*_3_-edge spectral profile obtained from the high-resolution partial fluorescence yield (PFY) model is an effective method to identify the valence state of Pb, we performed Pb *L*_3_-edge XAS using the PFY model^20,44,48^. As shown in Fig. 2b, a lower energy shoulder appeared at ~13,030 eV, which is assigned to the dipole-allowed transition from the 2*p*_3/2_ core level to the unoccupied 6*s* state. Typically, this pre-edge can be observed in Pb^4+^ ions with two 6*s* holes but is absent in Pb^2+^ ions with fully occupied 6*s* states^49^. Therefore, we can confirm that a Pb^4+^ component exists in PbFeO_3_. Meanwhile, we observed that the pre-edge peak height of PbFeO_3_ was lower than that of PbNiO_3_, which possesses a pure Pb^4+^ state. After subtracting the background originating from the edge jump and the Pb 5*d* states, the spectral intensity of the pre-edge peak (see the green curve in Fig. 2b) in PbFeO_3_ is approximately half of that in PbNiO_3_, indicating that the average valence state of Pb is +3. Moreover, the valence state of Pb can be further confirmed by HAXPES measurements. Figure 2c shows the HAXPES results for PbFeO_3_ and other Pb*M*O_3_ compounds with *M* = Ti, Cr, Co, and Ni used as standard references. Two components appeared in both the Pb 4*f*_7/2_ and Pb 4*f*_5/2_ peaks for PbCrO_3_ (Pb^2+^_0.5_Pb^4+^_0.5_CrO_3_), PbCoO_3_ (Pb^2+^_0.25_Pb^4+^_0.75_CoO_3_), and PbFeO_3_. Each peak can be deconvoluted into two Gaussians, as reported previously^18,19^. The 6*s*^0^ electronic configuration in Pb resulted in lower binding energy than that of 6*s*^2^ because of a strong screening effect^19^; hence, the components at lower binding energies are attributable to Pb^4+^ ions. The peak energies of PbFeO_3_ were close to those of Pb^2+^_0.5_Pb^4+^_0.5_Cr^3+^O_3_, indicating the coexistence of Pb^2+^ and Pb^4+^ ions. We estimated the fractions of Pb^2+^ and Pb^4+^ from the area ratios of Pb^2+^ and Pb^4+^ using PbCrO_3_ data as the standard for Pb^2+^_0.5_Pb^4+^_0.5_. As shown in Fig. 2d and Supplementary Table S3, the fitting demonstrates a nearly equal ratio between Pb^2+^ and Pb^4+^ in PbFeO_3_. Based on the BVS, XAS, and HAXPES results, we confirm that the charge configuration of PbFeO_3_ is Pb^2+^_0.5_Pb^4+^_0.5_Fe^3+^O_3_ with novel ordered Pb^2+^ and Pb^4+^ distribution.

### Electrical transport property of PbFeO_3_

**Fig. 3:**
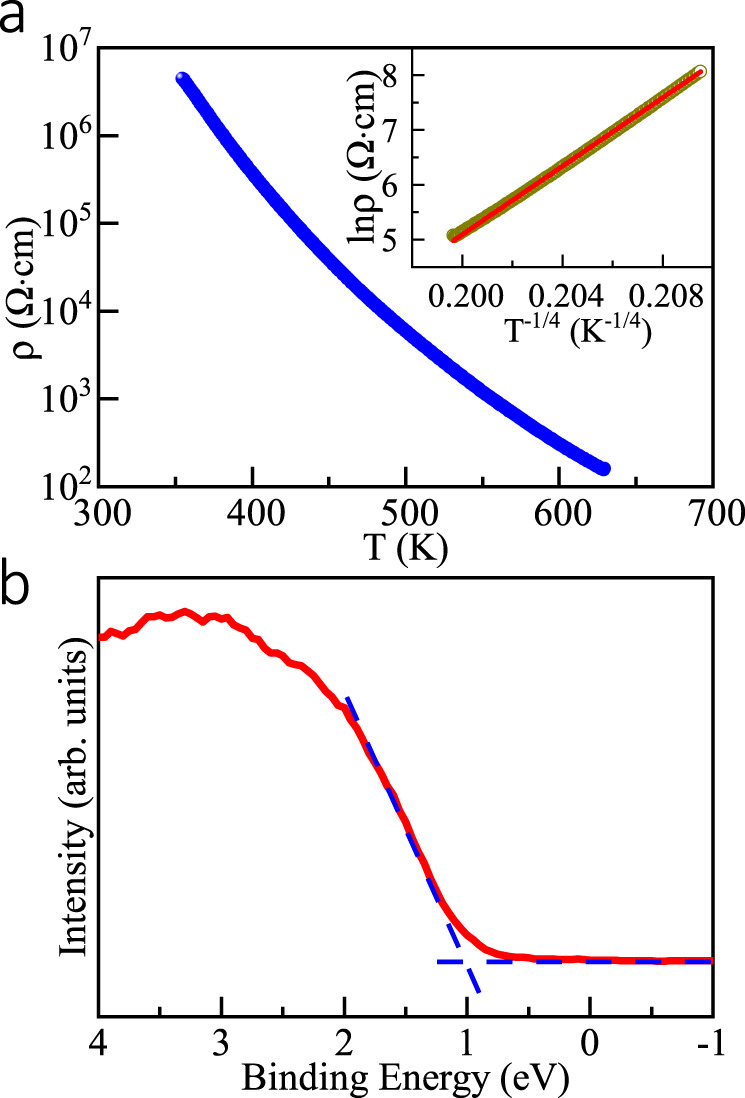
Electrical properties of PbFeO_3_. **a** Temperature dependence of resistivity measured above RT for PbFeO_3_. Inset shows 3D VRH model fitting between 520 and 630 K. **b** HAXPES spectrum near valence band for PbFeO_3_. The energy bandgap is evaluated to be ~1 eV.

The electrical properties of PbFeO_3_ are shown in Fig. 3a. A strong insulating feature was observed below 350 K (resistivity *ρ* > 10^7^ Ω ∙ cm), and a temperature-dependent behavior appeared with the values reduced by four to five orders of magnitude in the temperature range of 350–630 K. The data can be described by a three-dimensional (3D) variable-range-hopping (VRH) regime with the function *ρ*(*T*) = *ρ*_0_exp(*T*_0_/*T*)^1/4^, where *T*_0_ = (3^3^/π)/*k*_B_*ξ*^2^*D*(*E*_F_), *k*_B_ is the Boltzmann constant, *D*(*E*_F_) the density of states (DOS) at the Fermi level, and *ξ* the localization length of a wave function for localized electrons [exp(-r/*ξ*)]^50,51^. The inset of Fig. 3a shows the plot of ln*ρ* vs. *T*^−1/4^ for the resistivity data of PbFeO_3_, and the straight line of the plot is the fitting result obtained using the VRH model in the temperature range of 520–630 K. The fitting results yielded *T*_0_ = 5414 K, and *ρ*_0_ = 2.0 × 10^−4^ Ω ∙ cm. This implies that in this system the electrical transport is dominated by the hopping of localized charge carriers. This feature is consistent with the intrinsic insulating behavior of the Fe^3+^O_6_ perovskite framework, such as LaFeO_3_ and BiFeO_3_, owing to the strong electronic correlation effects^47,52^. Moreover, the valence band-edge positions obtained from HAXPES indicate that the valence bond is away from the Fermi energy and the binding energy is ~1 eV (see Fig. 3b), consistent with the insulating nature of PbFeO_3_. It is noteworthy that no electrical anomaly was observed at the magnetic transition temperatures as shown later.

### Electronic structures and charge-order analysis by DFT

**Fig. 4:**
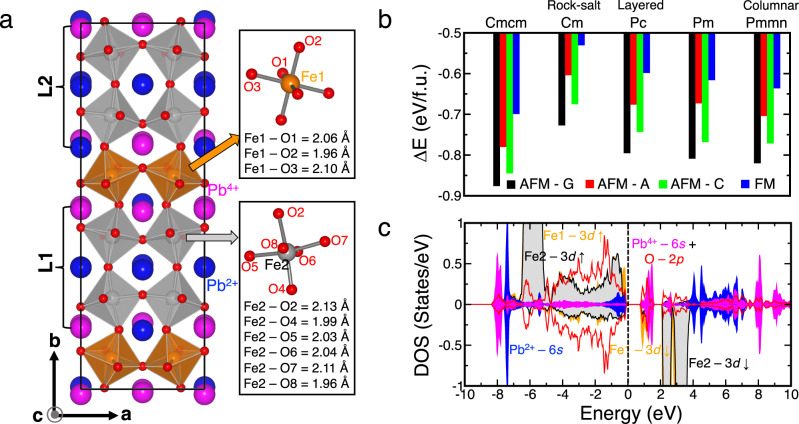
Charger-ordered phase description and electronic structure analysis. **a** Description of Pb^2+^/Pb^4+^-ordered optimized *Cmcm* structure. **b** Calculated energies of various Pb^2+^/Pb^4+^-ordered structures considering four collinear arrangements between Fe spins. Relative energy (${\Delta}E$) is shown with respect to undistorted ferromagnetic phase. **c** Calculated density of states of lowest energy charge-ordered *Cmcm* structure in *G*-type antiferromagnetic phase.

The crystal and electronic structures of the extraordinary charge-ordered states of Pb^2+^ and Pb^4+^ were further investigated by employing the DFT calculations for PbFeO_3_. To gain a deeper insight, we conducted a comparative analysis of the experimentally observed charge-order structure and other typically known *A*-site charge-ordered phases. We optimized the crystal structure of various Pb^2+^/Pb^4+^ 1:1 charge-ordered phases (see Supplementary Fig. S4) considering several collinear FM and AFM arrangements of Fe spins. The corresponding results are summarized in Fig. 4. We discovered that the experimentally determined *Cmcm* Pb^2+^/Pb^4+^ charge-order phase corresponded to the lowest energy structure and was 56 meV/f.u., which was lower compared with the nonpolar columnar-ordered *Pmmn* structure (see Figs. 4a and 4b). All the polar phases (*Cm*, *Pc*, and *Pm* shown in Supplementary Fig. S4), which were considered in this study, had higher energies compared with their nonpolar counterparts. The existence of two different types of lead ions in the *Cmcm* structure was reflected in our analysis of the calculated density of states (see Fig. 4c). The conduction bands within the energy range of the Fermi level to 2 eV comprised Pb 6*s* orbitals that were strongly hybridized with O 2*p* orbitals. This implies that within the *Cmcm* structure, half of the Pb ions were highly valent (+4), similar to several other Pb-based materials^16,53^. The other half of the Pb ions formed a 6*s*^2^ electronic structure, which corresponds to the +2 valence state. Consequently, the Fe ions exhibited half-filled *t*_2g_^3^*e*_g_^2^ electronic structures, thereby resulting in a strong AFM interaction between the Fe spins, which stabilized the *G*-type AFM structure (see Fig. 4b). The *G*-type AFM phase was insulating and indicated a bandgap of ~0.8 eV, agreeing with the experimentally determined value.

Various lattice distortions contributed to the formation of the charge-order *Cmcm* structure. As shown in Fig. 4a, the Pb charge-ordered patterns lead to the formation of layered-like components (denoted as **L1** and **L2**) made up of Fe2 ions. A layer of Fe1 ions remains sandwiched between every such **L1** and **L2** layers. We can categorize these distortions that contribute to the nonpolar *Cmcm* structure as follows. First, based on the oxygen octahedral rotations around the crystallographic axes, we have identified, (a) in-phase oxygen octahedral rotations around the crystallographic *c* axis, which correspond to the $M_3^ +$ symmetry of the undistorted and charge disordered cubic $Pm\overline 3 m$ structure, (b) out-of-phase oxygen octahedral rotations around the *b* axis (corresponding to the $R_4^ +$ symmetry of the cubic $Pm\overline 3 m$ structure), and (c) in-phase oxygen octahedral rotations around the *a* axis around the Fe1 ions (corresponding to the *Z*4 [1/3, 1/2, 0] symmetry of the cubic $Pm\overline 3 m$ structure), which can transform the cubic $Pm\overline 3 m$ structure to $Pmma$ structure. Next, we categorize the distortions that have been observed to occur exclusively around the Fe2 ions in the layered-like components (**L1** and **L2**) of the charge-order *Cmcm* structure in response to the non-trivial arrangement of the Pb^2+^ and Pb^4+^ cations (as illustrated in Supplementary Fig. S9). This category of distortions can be further classified as, (a) strong anti-ferro-distortive (AFD) movements of the oxygen and Fe2 ions in response to the difference in the charge states of the PbO layers along the *b* axis which correspond to the *DT*1 [0, 1/3, 0] symmetry (transform $Pm\overline 3 m \to P4/mmm$), (b) weak buckling in the O-Fe2-O connections along with the *a* and *c* axes (which follows the *DT*2 [0, 1/3, 0] symmetry transforming $Pm\overline 3 m \to Pmmm$), (c) AFD movements of the oxygens and Fe2 ions along the *a* and *c* axes which transform as *Z*4 [1/6, 1/2, 0] (transform $Pm\overline 3 m \to Cmmm$, hereafter denoted as *Z*4-I) and *Z4* [1/3, 1/2, 0] (transform $Pm\overline 3 m \to Pmma$, hereafter denoted as *Z*4-II) symmetries. The third category of distortions is the weak AFD movements of the Pb and oxygens ions following $R_5^ +$, $X_5^ +$, and $T3$ symmetries. The theoretically determined Fe–O bond lengths, as listed in Fig. 4a, agree well with their experimentally determined values (see Table 2). Furthermore, similar AFD phenomena were also exhibited by other layered and layered-like charge-order structures that were taken into consideration (Supplementary Fig. S4) in this study.

### Magnetic transitions and spin structures of PbFeO_3_

**Fig. 5:**
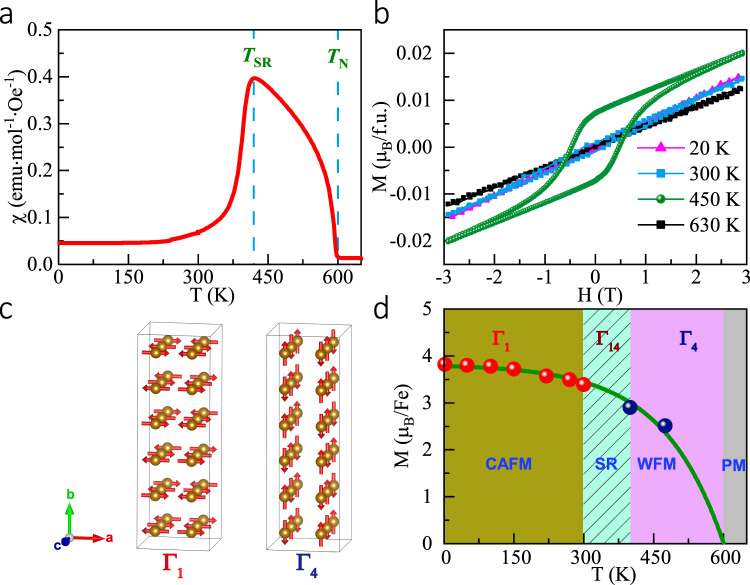
Magnetism measurements and schematic description of magnetic properties. **a** Temperature dependence of magnetic susceptibility of PbFeO_3_ measured at 0.01 T. **b** Isothermal magnetization loops measured at various temperatures. **c** Magnetic structures of PbFeO_3_ between *T*_SR_ and *T*_N_ (Γ_4_) as well as below 300 K (Γ_1_). Red arrows depict Fe^3+^ moments. **d** Magnetic-phase diagram for PbFeO_3_. Red and blue spots denote magnetic moments determined from neutron diffraction. The error bars of magnetic moments are within the symbols. Green line provides visual guidance. CAFM collinear antiferromagnetism, WFM weak ferromagnetism, PM paramagnetism.

The zero-field cooling (ZFC) magnetic susceptibility curve is illustrated in Fig. 5a. As the temperature decreased to *T*_N_ ≈600 K, the magnetic susceptibility increased significantly, indicating a long-range magnetic transition. Upon further cooling to *T*_SR_ ≈418 K, an abrupt decrease occurred, suggesting the presence of an SR transition. Based on the isothermal magnetization curves measured at different temperatures (see Fig. 5b), a linear magnetization behavior can be observed at 630 K, in accordance with paramagnetism. However, between *T*_N_ and *T*_SR_, considerable magnetic hysteresis occurred. For example, at 450 K, the observed coercive field was ~0.5 T. Moreover, there was a small amount of residual magnetization (0.01 *μ*_B_/Fe). These features indicate that a weak FM transition originating from the canted AFM spins would occur at *T*_N_. Below 300 K, the magnetization almost resumed to a linear behavior with negligible coercive field and residual magnetization, illustrating that the AFM structure did not comprise a weak ferromagnetic component. These behaviors are similar to those observed in *R*FeO_3_ perovskite oxides with a spin reorientation transition^33–35^. The Mössbauer spectrum at 300 K is shown in Supplementary Fig. S5. The spectrum can be fitted with three sets of magnetically split sextets with area ratios of 60, 34, and 6%. These components are attributed to Fe at the 16 *h* site, 8*d* site, and at *α*-Fe_2_O_3_ impurities. The values of the isomer shift and magnetic hyperfine field for the 16 *h* component (0.42 mm/s, 48.8 T) and those for the 8*d* component (0.38 mm/s, 42.0 T) indicate that Fe in PbFeO_3_ is trivalent and in a magnetically ordered state at RT.

To further determine the spin ordering of PbFeO_3_, we performed temperature-dependent NPD measurements from 2 to 625 K, as shown in Supplementary Figs. S6, S7, and S8. Supplementary Fig. S6 shows the diffraction patterns together with the Rietveld refinement results at 100 and 475 K. The Rietveld analysis for magnetic diffraction revealed a commensurate magnetic order with wave vector **k** = (0, 0, 0). Furthermore, a canted *G*-type AFM structure described by Γ_4_ (*G*_*x*_, *A*_*y*_, *F*_*z*_) (Bertaut’s notation^54^) with antiferromagnetically coupled spins along the *a* axis and an allowed net magnetization along the *c* axis was determined between *T*_SR_ and *T*_N_. The weak FM component had likely contributed to the magnetic hysteresis observed in the M–H measurement at 450 K; however, it was extremely small to be determined by the refinement. When the temperature was below *T*_SR_, a collinear *G*-type AFM structure described by Γ_1_ (*A*_*x*_, *G*_*y*_, *C*_*z*_) with spin along the *b* axis was also confirmed by the refinement, consistent with the linear feature of the magnetization shown in Fig. 5b. It is apparent that the symmetry of Γ_1_ (*A*_*x*_, *G*_*y*_, *C*_*z*_) forbids a FM component, which is also consistent with the absence of magnetic hysteresis below *T*_SR._ Supplementary Fig. S7 shows the NPD fitting for several characteristic peaks with the spin direction along *a* axis (Γ_4_), *b* axis (Γ_1_), and *c* axis (Γ_2_) for comparison. It is evident that the Γ_4_ and Γ_1_ spin models can provide the most reliable fitting for the higher and lower temperature magnetic phases, respectively. Therefore, our studies confirmed a temperature-induced SR from Γ_4_ (*G*_*x*_, *A*_*y*_, *F*_*z*_) to Γ_1_ (*A*_*x*_, *G*_*y*_, *C*_*z*_) in PbFeO_3_. Figure 5c shows the two different types of spin configurations. The refined magnetic moments at *T* = 475 and 100 K in units of *µ*_B_ were ***M***_*a*_ = 2.57 (2) and ***M***_*b*_ = 3.78 (1), respectively. Figure 5d shows the magnetic-phase diagram of PbFeO_3_ based on the current results. With decreasing temperature, the compound changed from paramagnetic to canted AFM with a small amount of net FM moment at the critical temperature of *T*_N_ ≈600 K. Upon further cooling to *T*_SR_ ≈418 K, a continuous SR transition occurred, changing the weak FM phase to a collinear AFM phase. The evaluated temperature range for the SR was between 300 and 418 K. Temperature-dependent magnetic moments for various phases are also shown in the phase diagram. Based on the refinement, the magnetic moment at *T* = 2 K in units of *µ*_B_ was ***M***_*b*_ = 3.8 (1), which was somewhat lower than the expected value (5 *µ*_B_) for an Fe^3+^ ion probably due to the considerable covalence/hybridization effects between Fe and O atoms (see Fig. 5c).

### Possible origins of spin reorientation in PbFeO_3_

In Fe-based perovskite oxides *R*FeO_3_^33,34,55^, the anisotropic *R*–Fe magnetic interactions are generally identified as driving factors behind the SR transitions. In the PbFeO_3_ system under investigation, the nonmagnetic nature of the *A*-site Pb^2+^ and Pb^4+^ ions negates the possibility of magnetic interactions between *A*- and *B*-site cations. However, PbFeO_3_ still exhibits a SR transition at a much higher critical temperature than those observed in most *R*FeO_3_ perovskites. This indicates that distinct mechanisms of SR transition are operational in PbFeO_3_. We further explored this phenomenon by the application of first-principles electronic structure calculations and finite temperature Monte Carlo simulations. Our investigations are based on the spin Hamiltonian as described below Eq. (1),

1 $${\cal{H}} =	 \,\mathop {\sum}\nolimits_{n,n^{\prime} } {J_{nn^{\prime} }^{11}} {\mathbf{S}}_n^1 \cdot {\mathbf{S}}_{n^{\prime} }^1 + \mathop {\sum}\nolimits_{n,m} {J_{nm}^{12}} {\mathbf{S}}_n^1 \cdot {\mathbf{S}}_m^2 + \mathop {\sum}\nolimits_{m,m^{\prime} } {J_{mm^{\prime} }^{22}} {\mathbf{S}}_m^2 \cdot {\mathbf{S}}_{m^{\prime} }^2\\ \,	 + \mathop {\sum}\nolimits_n {[D^1\left( {{\mathbf{S}}_{nz}^1} \right)^2 + E^1\{ \left( {{\mathbf{S}}_{nx}^1} \right)^2 - \left( {{\mathbf{S}}_{ny}^1} \right)^2\} ]}\\ \,	+ \mathop {\sum}\nolimits_m {[D^2\left( {{\mathbf{S}}_{mz}^2} \right)^2 + E^2\{ \left( {{\mathbf{S}}_{mx}^2} \right)^2 - \left( {{\mathbf{S}}_{my}^2} \right)^2\} ]}$$

Here, the first, second, and third terms denote the symmetric exchange interactions between individual Fe spins (${\mathbf{S}}^1$ and ${\mathbf{S}}^2$ denote Fe1 and Fe2 spin, respectively). The longitudinal and transverse anisotropy for the Fe1 and Fe2 ions are represented as ($D^1$ and $E^1$) and ($D^2$ and $E^2$), respectively. The magnetic interaction strengths estimated on the basis of the crystal structure experimentally determined at 300 K (denoted as S_Expt_) and the first-principles optimized structure (denoted as S_Opt_) are listed in Supplementary Fig. S10. The interactions between Fe spins, corresponding to either of these structures, are both strong and antiferromagnetic in nature leading to *G*-type AFM order. We also found that, while the Fe1 ions energetically favor the orientation of the spins along the *b* axis, the Fe2 spins, in contrast, favor the *a*-axis spin orientation. Moreover, the magnetic anisotropy of the Fe1 magnetic sublattice is stronger than that of the Fe2 sublattice. Our results of total energy calculations identified the *G*_*y*_ phase with spins parallel to the *b* axis to be the lowest energy phase for both the crystal structures, with energy of ~0.020 meV/Fe lower than that of the *G*_*x*_ phase with spins along the *a* axis. This is consistent with experimental observations. The Monte Carlo simulated magnetic transition temperature is around ~582 K (see Supplementary Fig. S11), which is also in good agreement with the experimentally observed *T*_N_. Below 582 K, both Fe1 and Fe2 sublattices exhibit *G*-type AFM ordering with their spins oriented along the *b* axis (see Supplementary Fig. S11). The spin Hamiltonian premised on S_Opt_ exhibits similar behavior (see Supplementary Fig. S11). Here, the anti-symmetric and anisotropic–symmetric exchange interactions between the Fe spins that lead to the spin canting were not taken into consideration, as these interactions, being weak for Fe^3+^ ions, are least likely to cause a spin orientation transition within a temperature interval of ~180 K.

**Fig. 6:**
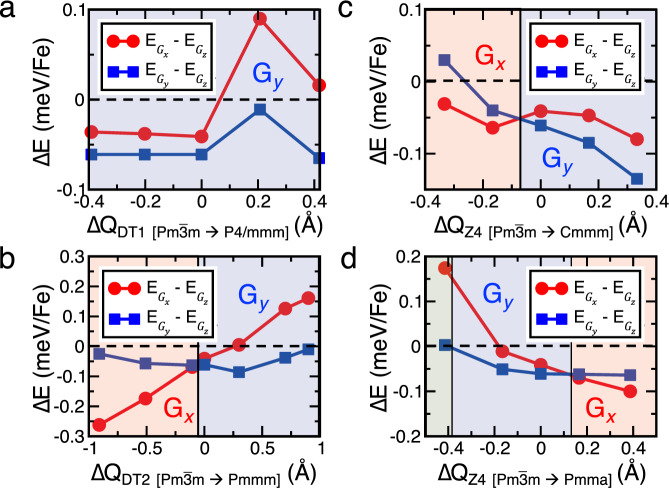
Effects of structural modulations on the stability of magnetic phases of PbFeO_3_. Calculated relative energies of *G*_*x*_, *G*_*y*_, and *G*_*z*_ magnetic phases as functions of the modulation of the structural distortions corresponding to the *DT*1 (transforms $Pm\overline 3 m \to P4/mmm$, **a**), *DT*2 (transforms $Pm\overline 3 m \to Pmmm$, **b**), *Z*4 (transforms $Pm\overline 3 m \to Cmmm$, **c**) and *Z*4 (transforms $Pm\overline 3 m \to Pmma$, **d**) symmetries with respect to the crystal structure experimentally determined at 300 K (S_Expt_).

The peculiar arrangement of the Pb ions leads to the creation of the two magnetic Fe1 and Fe2 sublattices with mutually competing magnetic anisotropic energies. We, therefore, explored the possible origin of SR transition through the nature of modulation of single-ion anisotropic energies. We calculated the total energies of the magnetic phases (*G*_*x*_, *G*_*y*_, and *G*_*z*_) as functions of the modulation of the structural distortions mentioned earlier. Among the various distortions in S_Expt_, only the symmetric distortions which owe their origin to the special arrangements of Pb cations, and eventually are transformed following *DT*1, *DT*2, and *Z*4 symmetries (see Supplementary Fig. S9), were taken into consideration. Our results (as shown in Fig. 6) indicate that an increase in the *DT*1 distortion results in the relative shortening of the average Fe1–O bond length along the *b* axis, which in turn enhances the magnetic anisotropy energies of Fe1 ions, contributing to the enhancement of stability of the *G*_*y*_ phase. This stability of the *G*_*y*_ phase is maintained within a range of ~0.8 Å modulation of the amplitude of this distortion with respect to S_Expt_. An increase in the *DT*2 and *Z*4-I distortions, which modulates the Fe2–O–Fe2 bond lengths and angles in the *ac* plane, also increases the stability of the *G*_*y*_ phase. The decrease in *DT*2 and *Z*4-I distortions, however, gives rise to the magnetic-phase transition from *G*_*y*_ to *G*_*x*_ phase (as shown in Fig. 6b, c). The same phase transition (i.e., from *G*_*y*_ to *G*_*x*_ phase) is also observed by increasing the AFD that follows the *Z*4-II symmetry along the *c* axis (see Fig. 6d). Interestingly, a decrease in the amplitude of this distortion below ~0.4 Å indicates a transition from the *G*_*y*_ to *G*_*z*_ phase. Our results are summarized as follows. (1) The *G*_*z*_ phase, in most cases, is higher in energy in comparison to the *G*_*x*_ and *G*_*y*_ phases. (2) *DT*2 and *Z*4-I are the two distortions that have been identified to contribute weakly to the formation of the *Cmcm* structure and significantly in the process of magnetic-phase transitions. Notably, the transformation of these distortions from zero to any finite value has no bearing on the overall symmetry of the structure. (3) Modulation of these distortions with respect to temperature can result in the transition from *G*_*x*_ to *G*_*y*_ magnetic phase. Notably, there is an exact match of the results between total energy calculations and Monte Carlo simulations based on estimated parameters of the Spin Hamiltonian as a function of *DT*2 and *Z*4-I structural distortions (as presented in Supplementary Fig. S12).

## Discussion

In summary, the crystal structure and physical properties of PbFeO_3_ were investigated in detail through SXRD, ED, XAS, HAXPES, NPD, and theoretical calculations. It was discovered that PbFeO_3_ possessed an unusual 2*a*_p_ × 6*a*_p_ × 2*a*_p_ orthorhombic superlattice with space group *Cmcm*. The BVS, XAS, HAXPES, and DFT calculation results revealed that the electronic configuration was Pb^2+^_0.5_Pb^4+^_0.5_Fe^3+^O_3_. A unique long-range charge ordering of the -A–B–B-type of the layers of Pb atoms with different oxidation states occurred. In the direction of the layers’ stacking, one layer with exclusively Pb^2+^ oxidation state (A) was followed by the two layers (B) with an average oxidation state of Pb^3.5+^, which in turn was a result of mixing 3×Pb^4+^ and 1×Pb^2+^ atoms in them. The single Pb^2+^ state formed two identically charge layers (A) at *y* ≈ 0 and 0.5, whereas the layers comprised of 3:1 ordered Pb^4+^ and Pb^2+^ atoms contributed to four other charge layers at the positions y ≈1/6, 1/3, 2/3, and 5/6. When the temperature decreased to 600 K, PbFeO_3_ experienced a canted AFM phase transition with a weak net FM moment. Further cooling induced a spin reorientation transition at ~418 K, thereby changing the canted AFM structure to a collinear one; the magnetic Fe moments were aligned along the *a* and *b* axes above and below the SR temperatures, respectively. The PbFeO_3_ system under investigation exhibits a unique phenomenon where two magnetic Fe1 and Fe2 sublattices are created as a result of the peculiar arrangement of Pb^2+^ and Pb^4+^ ions. The mutually competing magnetic anisotropic energies of these two sublattices is a plausible contributing factor for the spin reorientation in PbFeO_3_ at a higher critical temperature of 418 K. This is different from the case of *R*FeO_3_ perovskites where the presence of a magnetic rare-earth ion plays the most important role in the observed SR transitions. Since the magnetic sublattices owe their origin to a certain arrangement of Pb^2+^/Pb^4+^ ions, this may introduce a unique opportunity of inducing magnetic phase transition ($M = 0 \leftrightarrow M \ne 0$, where $M$ denotes net magnetization in the system) by driving a redistribution of Pb ions via an external electric field and/or strain. This work provides a new avenue for studying the charge ordering phase and distinctive spin orientation transition with potential applications in advanced spintronic devices due to the high transition temperature and possible tuning.

## Methods

### Materials synthesis

Polycrystalline samples of PbFeO_3_ were prepared using a high-pressure and high-temperature method. Starting materials of high purity (>99.9%) PbO, PbO_2_, and Fe_2_O_3_ with a 1:1:1 molar ratio were thoroughly mixed in an agate mortar within an argon-filled glovebox, and then sealed into gold capsules of diameter 3 mm and height 3.5 mm. The capsule was treated at 8 GPa and 1423 K for 30 min in a cubic-anvil-type high-pressure apparatus and quenched to room temperature prior to the slow release of pressure. The temperature window was narrow. Many impurities were introduced when the temperature increased or decreased by 50 K.

### Characterizations

The SXRD data for PbFeO_3_ were collected using a large Debye–Scherrer camera installed at the BL02B2 beamlines of SPring-8 with wavelengths of 0.41965 Å. The structure was refined using the Rietveld method via the GSAS program^56^. Temperature-dependent NPD at high temperatures (>300 K) was measured at HB-2A at the High Flux Isotope Reactor of the Oak Ridge National Laboratory^57^ with a wavelength of 2.41 Å. NPD patterns were obtained at ≤300 K using a high-resolution diffractometer HRPT^58^ at the Swiss Spallation Neutron Source of the Paul Scherrer Institute with a wavelength of 1.886 Å, and a vanadium container measuring 6 mm in diameter was used. The NPD data were analyzed using the Rietveld package, Fullprof^59^. The ED patterns and high-angle annular dark-field (HAADF) images at room temperature (RT) were obtained using a JEOL JEM-ARM200F (scanning) transmission electron microscope. The field dependence of the isothermal magnetization (*M*) and temperature-dependent magnetic susceptibility (*χ*) below 400 K were measured using a Quantum Design superconducting quantum interference device magnetometer. High-temperature magnetic susceptibility data at 400–650 K were collected using a MicroSense vibrating sample magnetometer. The resistivity at high temperatures was measured using the standard four-probe method. The size of the sample was ~2 × 1 × 1 mm for the electrical measurements. The valence states of PbFeO_3_ were determined via soft X-ray absorption spectroscopy and hard X-ray absorption near-edge spectroscopy (XANES). The XAS of Fe-*L*_2,3_ was collected at beamline BL11A of the National Synchrotron Radiation Research Center in Taiwan using the total-electron-yield mode. The spectrum of the single-crystal Fe_2_O_3_ sample was measured simultaneously to serve as an absolute energy reference. The high-resolution partial fluorescence yield Pb-*L*_3_ XAS spectra were measured at Pb *L*_1_ emission line with an overall resolution of ∼2 eV at beamline ID 20 of the Synchrotron SOLEIL, France. HAXPES measurements for Pb 4*f* core levels and valence bands were performed at RT with *E* = 7930.1 eV to investigate the valence state using a hemispherical photoelectron analyzer (R4000, VG Scienta) installed at BL09XU of SPring-8. Powder samples of Pb*M*O_3_ with *M* = Ti, Cr, Fe, and Ni were pasted onto a carbon tape. Carbon black powder was mixed with PbTiO_3_ before it was pasted onto a carbon tape to prevent charge-up due to the high insulation of the sample. The binding energy was calibrated using the Fermi edge of a gold film sample. Mössbauer spectroscopy was performed on a ^57^Fe-25%-enriched sample at RT using the conventional absorption method.

### First-principles calculations

The crystal and electronic structures of various Pb^2+^/Pb^4+^ charge-ordered states were studied by employing the DFT+$U$^60^ approach using the projector-augmented plane-wave method^61^ as implemented in the VASP code^62,63^. The Perdew–Burke–Ernzerhof (PBE)^64^ exchange-correlation functional was used. We constructed the initial structure of various *A*-site charge-ordered phases by freezing the anti-ferrodistortive displacements of oxygen ions and the oxygen octahedra rotations (which consequently created an *A*-site oxygen polyhedral with variation in size and oxygen coordination number) within the cubic $Pm\overline 3 m$ structure, which comprised eight chemical formula unit cell sizes. The corresponding structures were optimized considering several collinear arrangements of the Fe spins. We set the screened Coulomb interaction ($U$) to 4.5 eV and 1.0 eV for Hund’s coupling ($J_H$) on the Fe site. A kinetic-energy cutoff value of 500 eV and a Hellman–Feynman force convergence criterion of 0.01 eV Å^−1^ were used. A k-point mesh was considered based on the crystal symmetry. We further cross-checked the electronic structures of the charge-ordered states using the linearized augmented plane-wave (LAPW) method as implemented in the Wien2k code^65^ using the same values of $U$ and $J_H$ at the Fe site and k-point mesh, as used in the VASP code. We used the −7.5 Ry energy cutoff to separate the valence states from the core states. We calculated values of the symmetric exchange interactions between Fe spins and single-ion magnetic anisotropy energies of Fe1 ($E^1$ and $D^1$) and Fe2 ($E^2$ and $D^2$) ions by employing the linearized augmented plane-wave (LAPW) method. We estimated SIA parameters associated to Fe1 and Fe2 magnetic sublattices by modulating the lattice distortions which owe their origin to the special Pb^2+^/Pb^4+^ ordered pattern. The Monte Carlo simulations based on the first-principles parameterized spin Hamiltonian were conducted considering 8 × 4 × 8 (6144 magnetic ions) supercell size of the $Cmcm$ structure and maximum sample number of 10^9^.

## Supplementary information

Supplementary Information

Peer Review File

## Data Availability

The data that support the findings of this study are available from the corresponding authors upon reasonable request.
