# Supplementary material for: Observation of novel charge ordering and spin reorientation in perovskite oxide PbFeO3
Source: Nat Commun. 2021 Mar 26;12:1917. doi: 10.1038/s41467-021-22064-9 (PMC7997894; doi:10.1038/s41467-021-22064-9)
Supplement: Supplementary file 1 — Supplementary Information [file 41467_2021_22064_MOESM1_ESM.pdf]

Supplementary File for

Observation of novel charge ordering and spin reorientation in perovskite oxide $PbFeO_3$

Xubin Ye[1,2,#], Jianfa Zhao[1,2,#], Hena Das[3,4,#], Denis Sheptyakov[5], Junye Yang[5], Yuki Sakai[6,3], Hajime Hojo[7], Zhehong Liu[1,2], Long Zhou[1,2], Lipeng Cao[1], Takumi Nishikubo[3], Shogo Wakazaki[3], Cheng Dong[1,2], Xiao Wang[8], Zhiwei Hu[8], Hong-Ji Lin[9], Chien-Te Chen[9], Christoph Sahle[10], Anna Efiminko[10], Huibo Cao[11], Stuart Calder[11], Ko Mibu[12], Michel Kenzelmann[5], Liu Hao Tjeng[8], Runze Yu[*,1,2,4], Masaki Azuma[*,3,6], Changqing Jin[1,2,13], and Youwen Long[*,1,2,13]

[1]Beijing National Laboratory for Condensed Matter Physics, Institute of Physics, Chinese Academy of Sciences, Beijing 100190, China

[2]School of Physical Sciences, University of Chinese Academy of Sciences, Beijing 100049, China

[3]Laboratory for Materials and Structures, Tokyo Institute of Technology, 4259 Nagatsuta, Midori, Yokohama 226-8503, Japan

[4]Tokyo Tech World Research Hub Initiative (WRHI), Institute of Innovative Research, Tokyo Institute of Technology, 4259 Nagatsuta, Midori-ku, Yokohama, Kanagawa 226-8503, Japan

[5]Laboratory for Neutron Scattering and Imaging, Paul Scherrer Institut, CH-5232 Villigen, Switzerland

[6]Kanagawa Institute of Industrial Science and Technology, 705-1 Shimoimaizumi, Ebina 243-0435, Japan

[7]Department of Advanced Materials and Engineering, Faculty of Engineering Sciences, Kyushu University, Kasuga 816−8580, Japan

[8]Max-Planck Institute for Chemical Physics of Solids, Nöthnitzer Straße 40, 01187 Dresden, Germany

[9]National Synchrotron Radiation Research Center, 101 Hsin-Ann Road, Hsinchu 30076, Taiwan, R.O.C

[10]European Synchrotron Radiation Facility, 71 Avenue des Martyrs, 38043 Grenoble, France

[11]Neutron Scattering Division, Oak Ridge National Laboratory, Oak Ridge, Tennessee 37831, United States

[12]Graduate School of Engineering, Nagoya Institute of Technology, Nagoya 466-8555, Japan

[13]Songshan Lake Materials Laboratory, Dongguan, Guangdong 523808, China

[#]These authors contribute equally to this work.

*Corresponding email: ywlong@iphy.ac.cn (Y.W.L.); yurz@iphy.ac.cn (R.Z.Y.); and mazuma@msl.titech.ac.jp (M.A.)

Supplementary Table S1 | Crystallographic parameters of $PbFeO_3$ refined from SXRD pattern at RT[a]

atom	site	*x*	*y*	*z*	100×B_{iso} (Å)
Pb1	4*c*	0	-0.0037(1)	0.25	0.78(1)
Pb2	4*c*	0	0.4996(1)	0.25	0.78(1)
Pb3	4*c*	0	0.8282(1)	0.25	0.78(1)
Pb4	4*c*	0	0.1546(1)	0.25	0.78(1)
Pb5	4*c*	0	0.3349(1)	0.25	0.78(1)
Pb6	4*c*	0	0.6593(1)	0.25	0.78(1)
Fe1	8*d*	0.25	0.25	0	0.22(2)
Fe2	16*h*	0.2558(6)	0.5840(1)	0.9993(6)	0.22(2)
O1	8*g*	0.301(4)	0.7756(8)	0.25	1.8(3)
O2	16*h*	0.308(2)	0.6732(7)	0.053 (2)	1.8(3)
O3	8*f*	0	0.2703(9)	-0.064(4)	1.8(3)
O4	8*e*	0.277(4)	0	0	1.8(3)
O5	8*f*	0	0.0763(10)	1.050(4)	1.8(3)
O6	8*g*	0.187(4)	0.5869(8)	0.25	1.8(3)
O7	8*f*	0	0.6082(9)	-0.038 (4)	1.8(3)
O8	8*g*	0.283(4)	0.4047(7)	0.25	1.8(3)

[a]Space group *Cmcm* (No. 63), Z = 24. a = 7.88504(2)Å, b = 23.37741(8) Å, c = 7.70905(2) Å, ρ_{cal} = 8.723(1) g/cm^3, V = 1421.023(1) Å^3. R_{wp} = 7.63%, R_p = 5.30%, χ^2 = 9.20.

Supplementary Table S2 | Pb-O and Fe-O bond lengths and BVSs for $PbFeO_3$ Refined from SXRD pattern at RT.

	Distance (Å)		Average bond length (Å)	BVS
Fe1-O	1.897(2)	× 2	2.014	3.03
	2.058(2)	× 2		
	2.087(3)	× 2		
Fe2-O	1.952(3)		2.032	2.87
	1.973(3)			
	1.981(2)			
	2.009(3)			
	2.115(2)			
	2.165(2)			
Pb1-O	2.427(4)	× 2	2.771	1.94
	2.738(6)	× 2		
	2.866(6)	× 4		
	2.912(6)	× 2		
Pb2-O	2.516(4)	× 2	2.778	2.01
	2.611(5)	× 4		
	3.005(6)	× 2		
	3.148(6)	× 2		
Pb3-O	2.475(5)	× 2	2.774	2.23
	2.675(5)	× 2		
	2.712(6)	× 2		
	2.786(3)	× 4		
	3.213(3)	× 2		
Pb4-O	2.189(4)	× 4	2.588	3.72
	2.395(5)	× 2		
	2.933(4)	× 2		
	3.235(6)	× 2		
Pb5-O	2.092(4)	× 2	2.455	3.93
	2.107(4)	× 2		
	2.767(6)	× 2		
	2.853(5)	× 2		
Pb6-O	2.182(4)	× 2	2.541	3.52
	2.245(6)	× 2		
	2.521(4)	× 2		
	2.880(4)	× 4		

Supplementary Table S3 | Peak area ratio for each peak and calculated average Pb valence for HAXPES data.

	$4f_{5/2}$		$4f_{7/2}$	
$PbCrO_3$	0.61	0.39	0.61	0.39
$PbFeO_3$	0.59	0.41	0.58	0.42
$PbCoO_3$	0.48	0.52	0.45	0.55

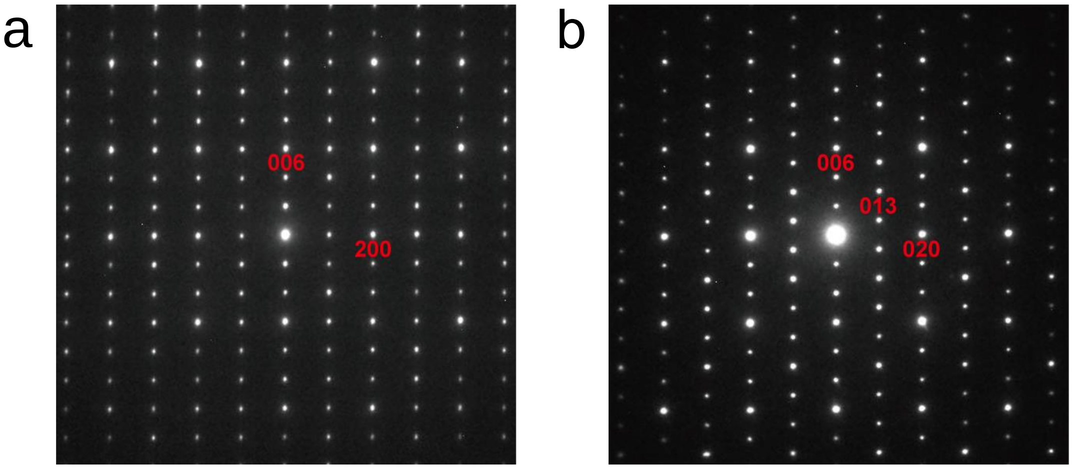

Supplementary Figure S1 | ED patterns along the pseudocubic zone axis at RT for $PbFeO_3$. (a) [100] and (b) [001] axis. Multiple scattering effects make it possible to observe h00 reflections with h = odd at [010].

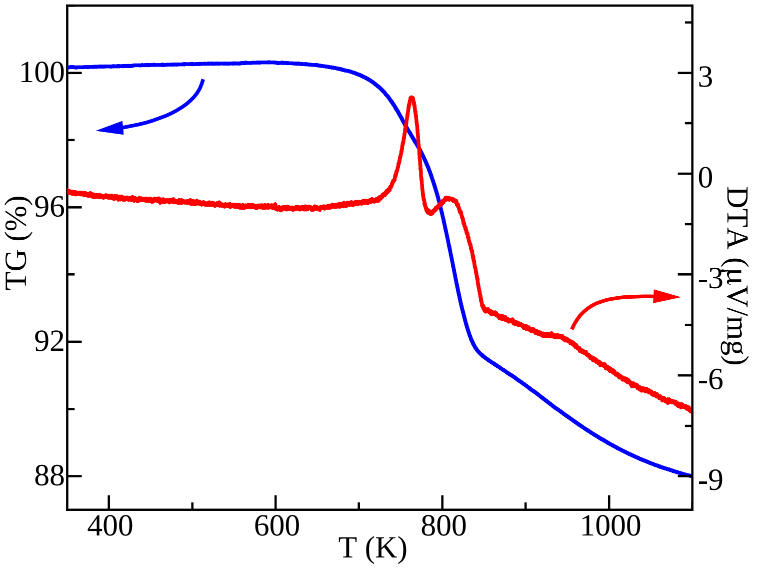

Supplementary Figure S2 | Temperature dependence of thermogravimetry (TG) and differential thermal analysis (DTA) for $PbFeO_3$.

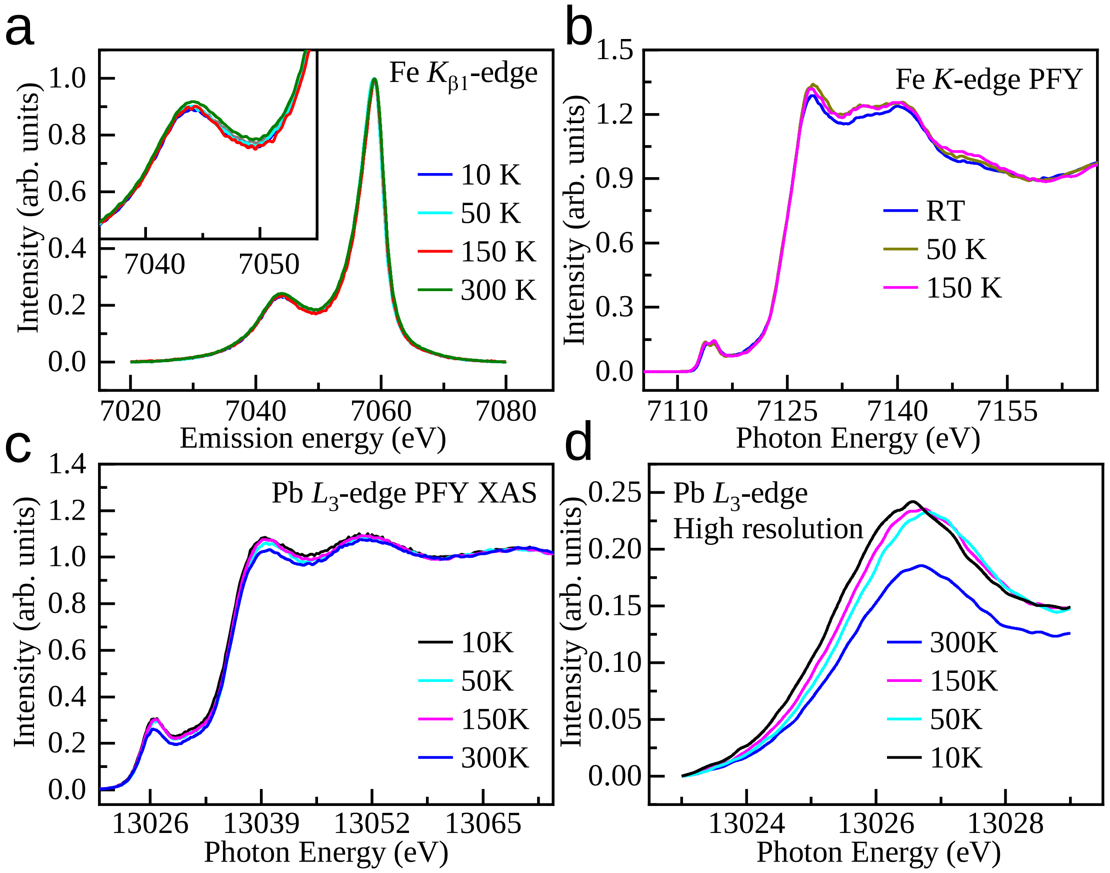

Supplementary Figure S3 | Temperature dependent XAS measurement for $PbFeO_3$. (a) Fe $K_{\beta1}$-edge for $PbFeO_3$. (b) Fe K-edge for $PbFeO_3$ in PFY model. (c) Pb L_3-edge for $PbFeO_3$ in PFY model. (d) High resolution Pb L_3-edge for $PbFeO_3$.

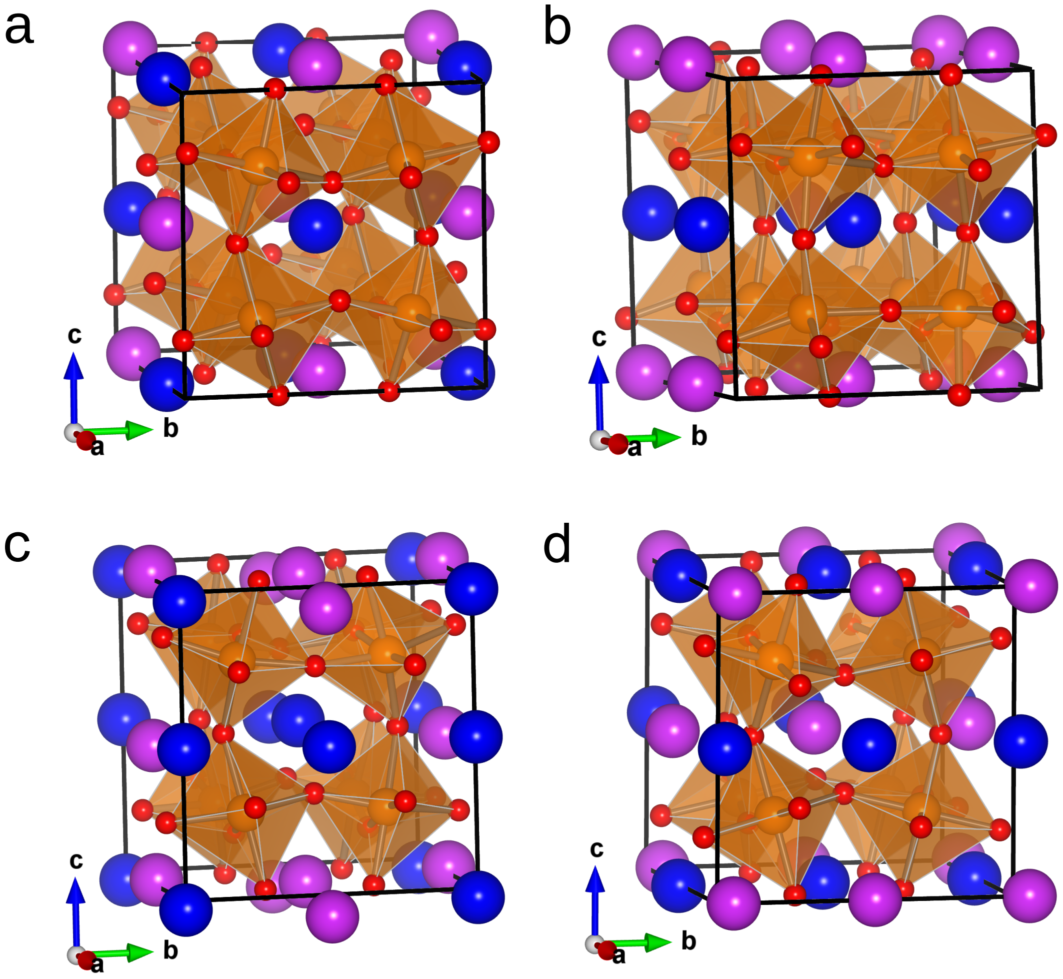

Supplementary Figure S4 | Represents the Pb^{2+}/Pb^{4+} for different charge-order phases. (a) rock-salt (*Cm*), (b) layered (*Pc*), (c) mixed (*Pm*) and (d) columnar (*Pmmn*).

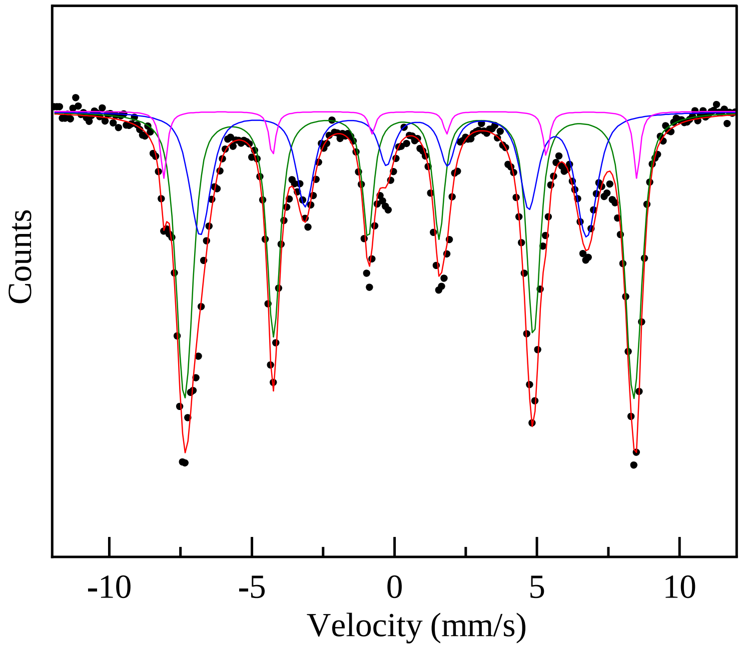

Supplementary Figure S5 | Mössbauer spectrum for the ^{57}Fe-enriched powder sample at 300 K. The spectrum can be fitted with three sets of magnetically-split sextets, which are attributed to Fe at the 16*h* site (olive, 60%), 8*d* site (blue, 34%), and at α-Fe_2O_3 impurities (pink, 6%).

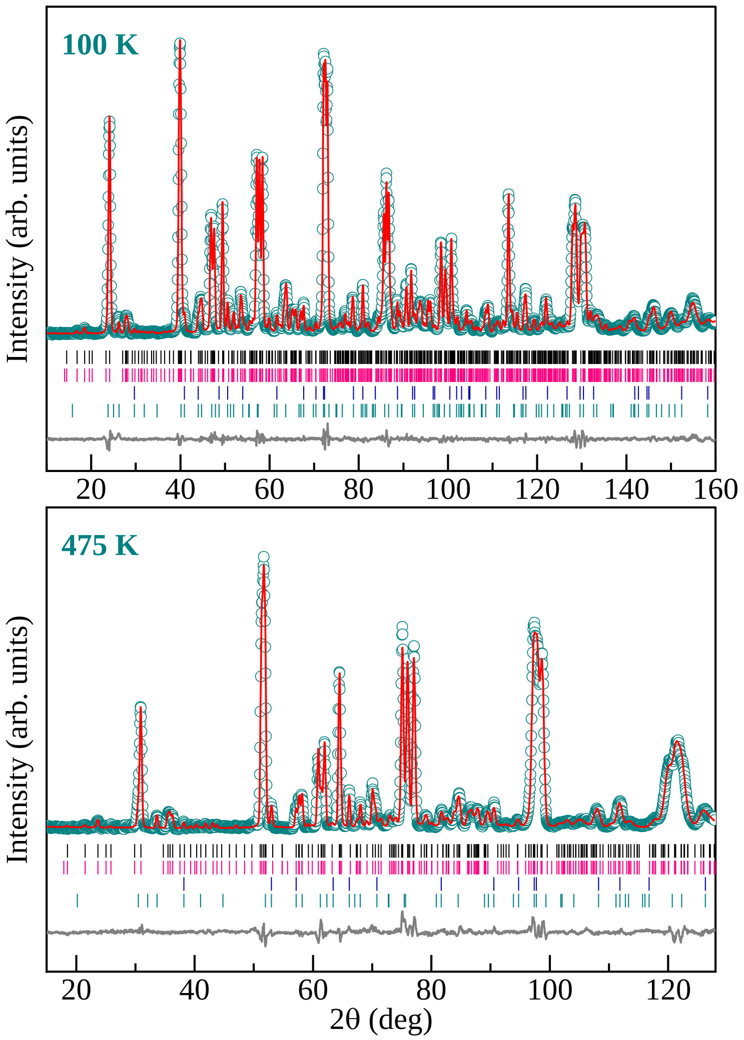

Supplementary Figure S6 | The NPD patterns and the refinement results at 100 K and 475 K for $PbFeO_3$. The observed (dark cyan circles), calculated (red line), and difference (dark gray) values are shown. The ticks correspond to the allowed nuclear (black), magnetic (pink) Bragg peaks of $PbFeO_3$ and allowed nuclear (navy), magnetic (green) Bragg reflections of the impurity phase Fe_2O_3 (~5 wt %), respectively.

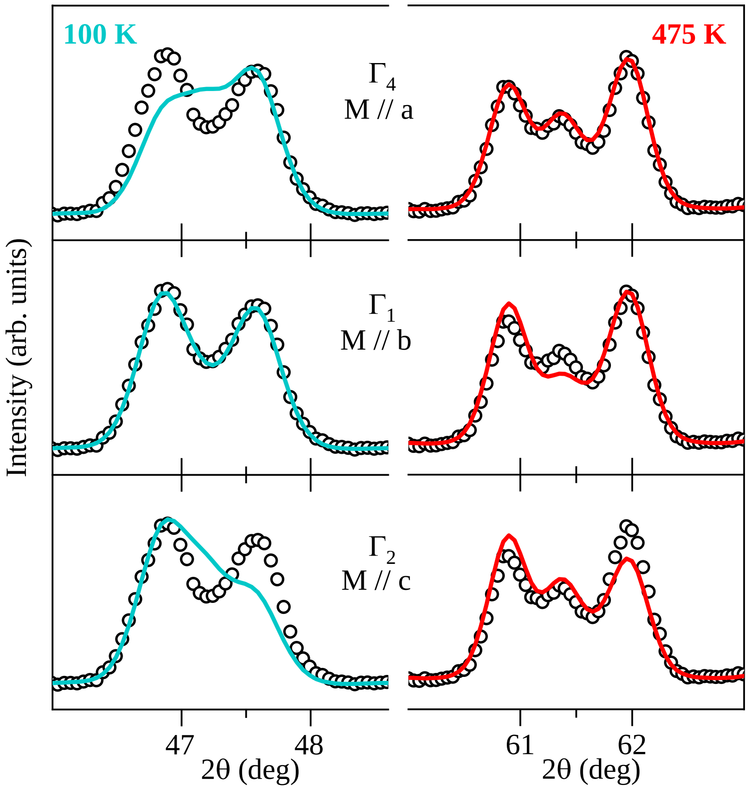

Supplementary Figure S7 | Magnetic Bragg peaks and the fitting curves using the three different spin models at 100 K (left panel) and 475 K (right panel) for several characteristic peaks of NPD patterns of $PbFeO_3$. The gray balls and solid curves correspond to measured data and fitted results, and three spin models (Γ_4, Γ_1 and Γ_2) are represented from up to down, respectively.

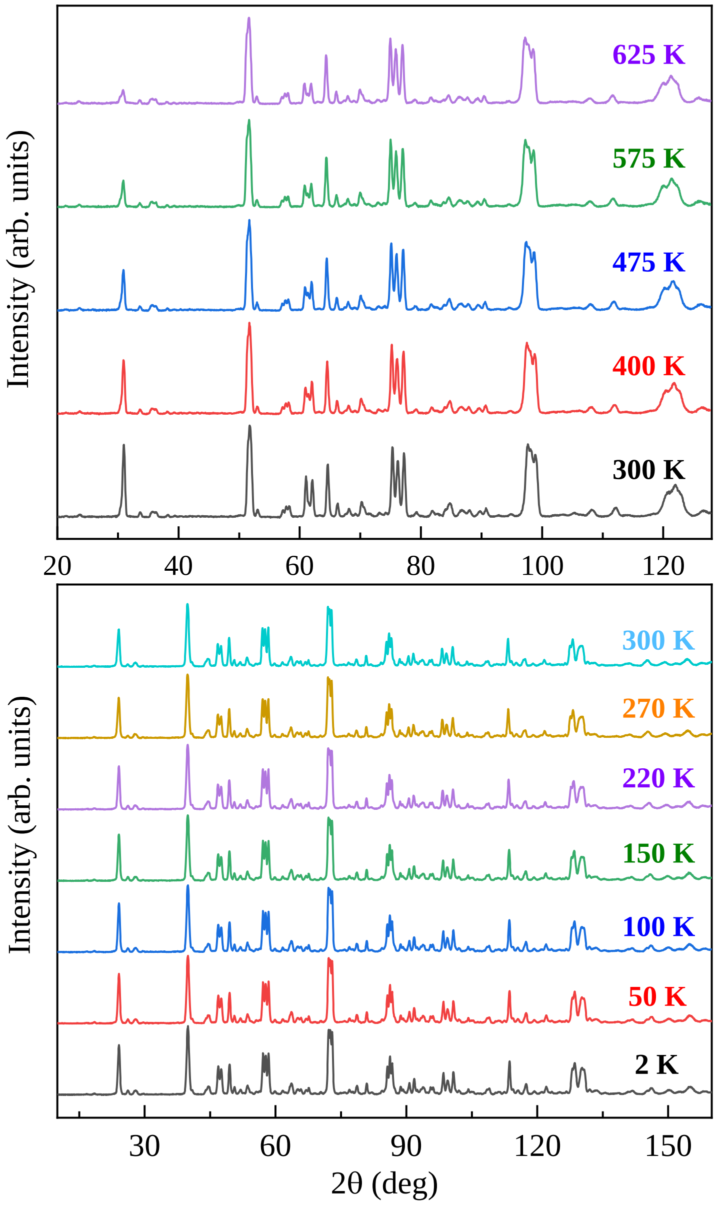

Supplementary Figure S8 | The NPD patterns measured at 2 - 625 K for $PbFeO_3$.

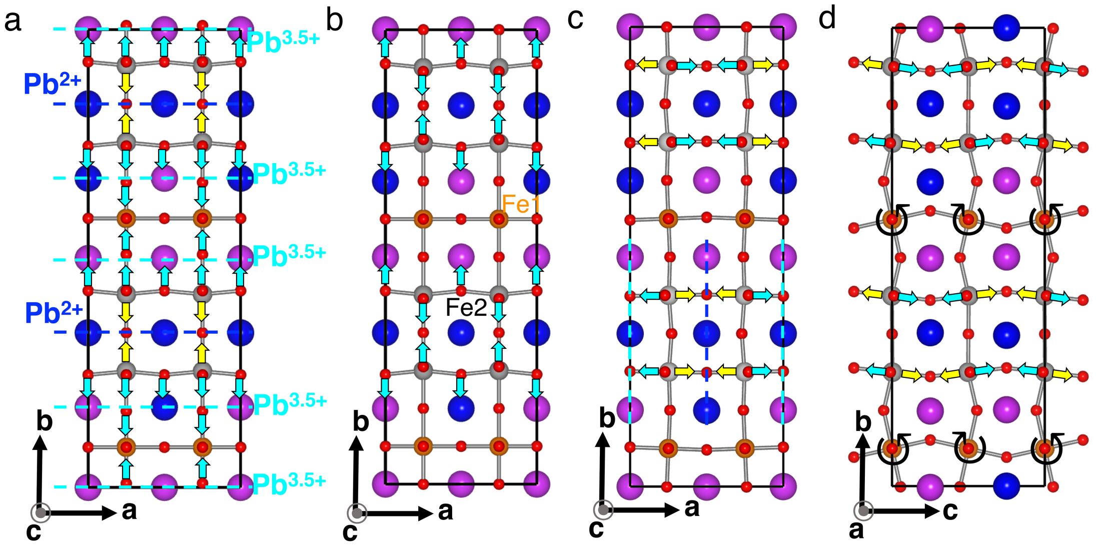

Supplementary Figure S9 | Symmetric distortions which owe their origin to the special arrangements of Pb cations. The symmetric distortions that correspond to $DT1$ (transforms $Pm\bar{3}m \rightarrow P4/mmm$, (a)), $DT2$ (transforms $Pm\bar{3}m \rightarrow Pmmm$, (b)), $Z4$ (transforms $Pm\bar{3}m \rightarrow Cmmm$, (c)) and $Z4$ (transforms $Pm\bar{3}m \rightarrow Pmma$, (d)). Blue and cyan layers represent average 2+ and 3.5+ oxidation state of Pb ions, respectively. The Fe and oxygen movements are denoted by yellow and cyan arrows, respectively. The in-phase oxygen octahedra rotations at the Fe1 layers around *a* axis is denoted by black arrow.

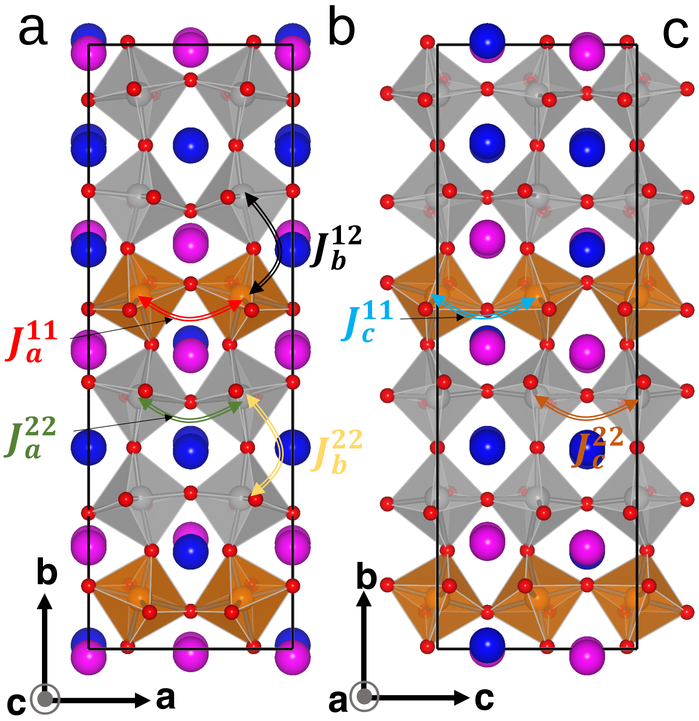

c

Magnetic exchange interactions in meV		
	Structure determined at 300 K	DFT optimized structure
J_a^{11}	4.129	4.018
J_c^{11}	3.935	4.197
J_b^{12}	5.080	5.189
J_a^{22}	5.312	5.212
J_c^{22}	6.787	6.559
J_b^{22}	7.971	7.877

SIA components in meV		
	Structure determined at 300 K	DFT optimized structure
E^1	0.021	0.023
D^1	0.019	0.016
E^2	-0.007	0.002
D^2	0.002	0.004

Supplementary Figure S10 | Parameterize the spin Hamiltonian. (a) and (b) Super-exchange interaction paths between Fe spins. (c) Estimated values of the symmetric exchange interactions between Fe spins and single ion magnetic anisotropy energies of Fe1 (E^1 and D^1) and Fe2 (E^2 and D^2) ions.

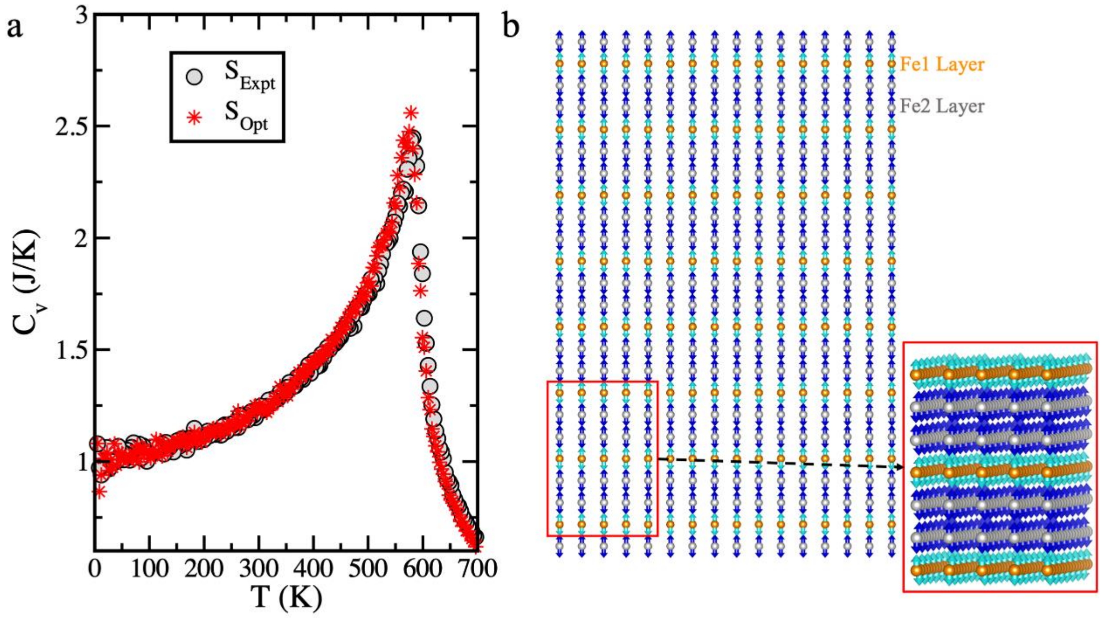

Supplementary Figure S11 | Results of finite temperature Monte Carlo simulations. (a) Exhibits magnetic phase transition ~ 580 K for both S_{Expt} and S_{Opt} structures. (b) Denotes the *G*-type (G_y) antiferromagnetic structure at 20 K. The cyan and blue arrows represent Fe1 and Fe2 spins, respectively.

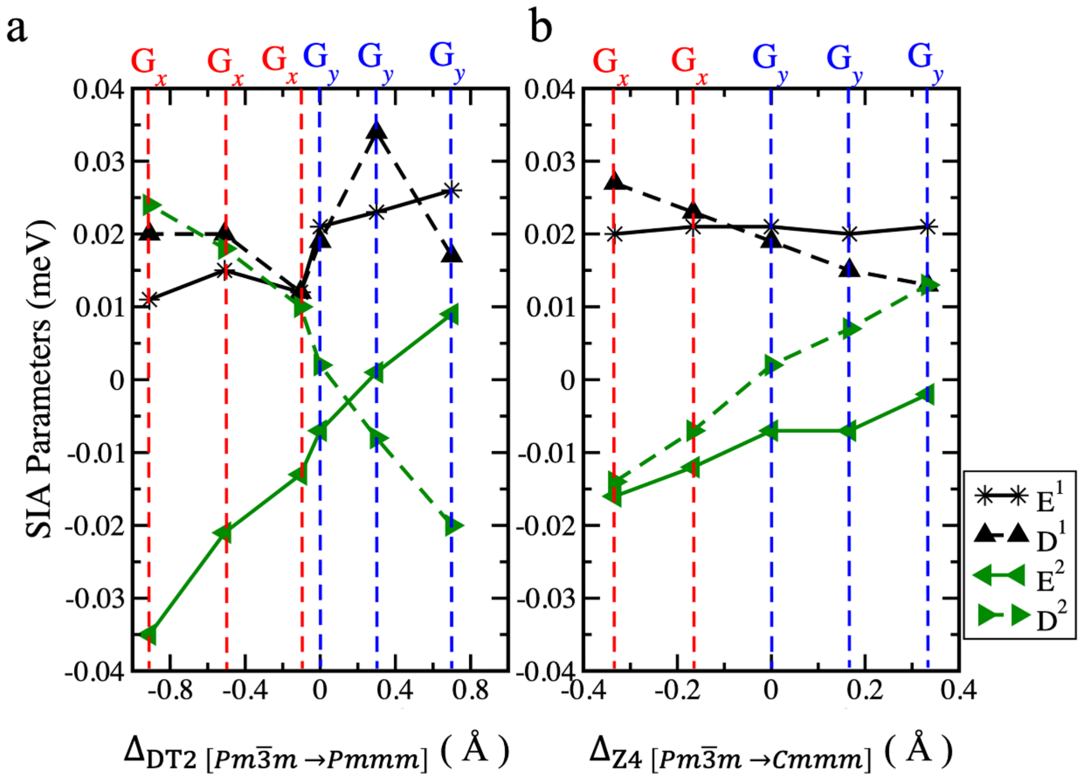

Supplementary Figure S12 | Stability of various magnetic phases as modulation of structural distortions. (a) and (b) Estimated single ion anisotropy (SIA) parameters associated with Fe1 (E^1 and D^1) and Fe2 (E^2 and D^2) magnetic sublattices as a function of $DT2$ ($Pm\bar{3}m \rightarrow Pmmm$) and $Z4$ ($Pm\bar{3}m \rightarrow Cmmm$) distortions, respectively. The magnetic phases stabilized in the Monte Carlo simulations considering the estimated SIA parameters below the magnetic phase transition are also shown.
